# A county-level spatial epidemiological study of hair selenium and Keshan disease

**DOI:** 10.3389/fnut.2022.1011460

**Published:** 2022-11-07

**Authors:** Yuehui Jia, Guijin Li, Ruixiang Wang, Chen Feng, Lei Qi, Yuanyuan Wang, Shengqi Su, Yuanjie Zou, Xu Liu, Yanan Wang, Yiyi Zhang, Linlin Du, Huixin Sun, Shuxiu Hao, Jie Hou, Hongqi Feng, Qi Li, Tong Wang

**Affiliations:** ^1^Institute of Keshan Disease, Chinese Center for Endemic Disease Control, Harbin Medical University, Harbin, China; ^2^School of Public Health, Qiqihar Medical University, Qiqihar, China; ^3^Institute of Environmental and Occupational Health, Ningbo Municipal Center for Disease Control and Prevention, Ningbo, China; ^4^Department of Emergency Management, Yidu Central Hospital of Weifang, Weifang, China; ^5^School of Public Health and Management, Binzhou Medical University, Yantai, China; ^6^Department of Food Hygiene, Yantai Disease Prevention and Control Center, Yantai, China; ^7^Department of Gynecological Radiotherapy, Harbin Medical University Cancer Hospital, Harbin, China

**Keywords:** Keshan disease, hair selenium, small area study, spatial autocorrelation analysis, precision assessment

## Abstract

**Background:**

No spatial analysis of hair selenium and Keshan disease (KD) on a nationwide county-level has been performed. Selenium deficiency is a recognized environmental risk factor for KD. Hair selenium is one of the recognized biomarkers of selenium nutrition. This study aimed to perform a geographically precise and visualized assessment of the achievement of KD prevention and control at the level of selenium nutrition in terms of etiology.

**Methods:**

A spatial ecological study was conducted. The hair selenium content of the residents was assayed using an atomic fluorescence spectrometer. The spatial analysis was performed using ArcGIS.

**Results:**

The median of the hair selenium levels of the 3,028 participants in the 1,174 counties was 0.38 mg/kg, and the content of inhabitants in KD endemic counties was significantly lower than that in KD non-endemic counties (0.34 vs. 0.39 mg/kg, *z* = −10.03, *P* < 0.0001). The proportion of Se-deficient and Se-marginal counties in KD endemic counties was significantly higher than that in KD non-endemic counties (59.4 vs. 29.0%, *z* = −7.45, *P* < 0.0001). The global autocorrelation analysis was not statistically significant (Moran's *I* = 0.0005, *P* = 0.68). Local autocorrelation analysis identified 174 low-low clusters of hair selenium levels, 83 (47.7%) of which are KD endemic counties located in KD endemic provinces of Henan, Gansu, Shaanxi, Inner Mongolia, Jilin, and Heilongjiang. The hair selenium featured a positive correlation with per capita GDP (*r*_s_ = 0.20, *P* < 0.0001).

**Conclusion:**

The median of the hair selenium levels of inhabitants living in KD endemic counties was significantly lower than that in KD non-endemic counties. All the 83 KD endemic counties with low-low clusters of hair selenium levels should be prioritized in KD precision prevention and control. These findings are geographically precise and visualized evidence of the assessment of the effectiveness of KD prevention and control at the level of selenium nutrition in terms of etiology.

## Introduction

Keshan disease (KD) is primary endemic cardiomyopathy that is strongly associated with selenium (Se) deficiency. KD endemic regions comprise 2,621 townships in 330 counties in 16 provinces in mainland China (1, 2). There are 62 million inhabitants living in the KD endemic townships. The etiological or risk indicators are of great significance for assessing the achievement of KD prevention and control.

Selenium is an essential trace element for human health (3–5). Studies fully confirmed that the occurrence of KD is strongly associated with selenium deficiency (6–10). The geographical distribution of KD endemic regions highly overlapped with the low-selenium geological belt (11–14). Therefore, the assessment of the selenium nutrition of inhabitants is an appropriate approach to evaluate the achievement of KD prevention and control in terms of etiology.

The selenium nutritional level for humans is mainly evaluated by measuring the selenium levels of blood, hair, urine, and nail samples (15–19). Whole blood selenium and hair selenium levels reflect a long-term (several weeks or even several months) selenium nutritional status and are the most common and widely used biomarkers for assessing selenium nutritional status for humans (20, 21). However, blood selenium is susceptible to dietary changes or acute pathologies. In contrast, hair selenium is highly stable and very suitable for selenium nutritional investigation. Measuring hair selenium has the advantages of being noninvasive, being convenient, having fast and safe sampling, and having easy storing of samples (22). Furthermore, because hair selenium level is highly correlated with the blood selenium level, it is being widely used (22). The reliability of urine and nail selenium has not been sufficiently studied. Furthermore, urine selenium is a more short-term biomarker (23).

A spatial epidemiological study is a very direct and effective way to precisely visualize the spatial distribution characteristics and clustering by mapping. Therefore, the spatial analysis can not only prioritize the keys of KD prevention and control but also assess their effectiveness (24–26). However, no spatial analysis of hair selenium and KD on a nationwide county level, which is a geographically precise and reliable small-area study, has been performed. The spatial epidemiological study at the county level was undertaken to describe and analyze the selenium nutritional status of inhabitants in KD endemic and non-endemic regions. From the etiological perspective, this not only provides geographically precise and visualized evidence for KD precision prevention and control at the county level but also assesses their effectiveness.

## Materials and methods

### Study design

**Figure 1:**
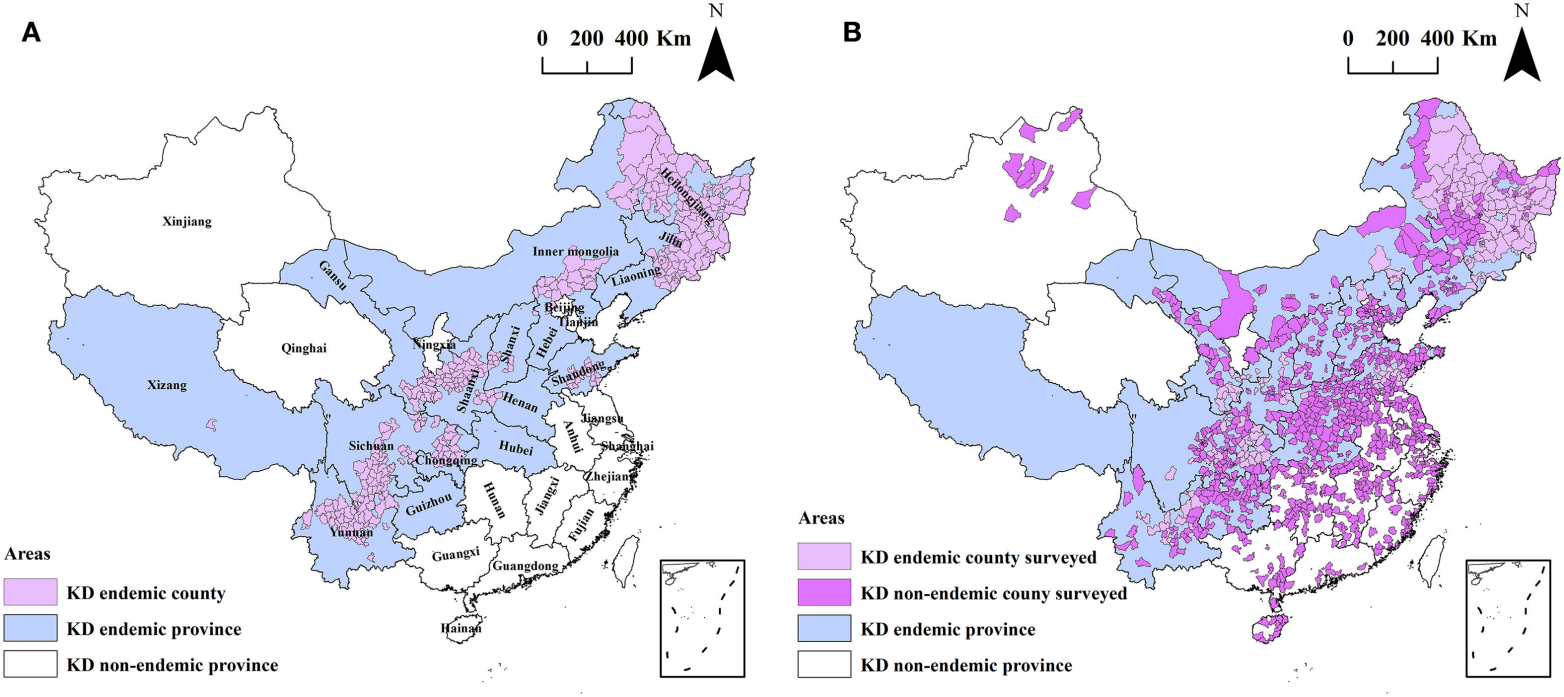
The spatial distribution of the KD endemic regions **(A)** and the surveyed counties in this study **(B)**.

During 2019–2020, a nationwide county-level spatial ecological study was designed to investigate hair selenium levels for 3,028 inhabitants in 1,174 counties across 28 provinces in mainland China. The spatial distribution of the KD endemic regions and the surveyed counties is shown in Figures 1A,B.

### Study participants

Participants had to meet the following inclusion criteria: (1) they were permanent male inhabitants that have lived in their residence for not <6 months in the recent 12 months, (2) they could communicate normally, (3) they provided written informed consent, (4) they had not permed or dyed their hair in the past 3 months, and (5) they had not used Se nutritional supplements for at least 3 months before the hair samples were collected.

### Criteria

The Keshan Disease Endemic Area Definition and Classification (27) indicates the delimitation of the KD endemic and non-endemic counties. The administrative divisions of China (28) indicates the delimitation of the cities, urban centers, and rural areas. The participants living in cities (counties) and districts of big cities are classified as city residents, those living in small urban towns are classified as urban residents, and those living in rural villages are classified as rural residents. In the classification standard of selenium for human hair in the Chinese Nutritional Science Encyclopedia, the hair selenium nutritional status of the participants are classified as Se-deficient (<0.2 mg/kg), Se-marginal (0.2 ≤ content < 0.36 mg/kg), Se-sufficient (0.36 ≤ content < 3.60 mg/kg), Se-rich (3.60 ≤ content ≤ 5.10 mg/kg), and Se-excessive (>5.10 mg/kg).

### Questionnaire survey

Participants were given a questionnaire including age, current residence and address, hair dyeing or perming status, and Se supplements.

### Hair samples

At least 1.0 g of hair samples were cut ~2 cm away from the skin. Subsequently, the collected hair samples were immediately packed into polyethylene bags and stored until the assay.

### Hair selenium

The cleaned and dried hair sample was weighed using a balance to 0.1 g, which was placed into a 50 ml digestion container. For quality control, the certified reference of national standard materials for human hair (GBW 07601a), containing selenium of 0.58 ± 0.12 mg/kg, were prepared in the same way as the hair samples. Then, 5 ml of mixed acid (concentrated nitric acid and per chloric acid blended in a ratio of 4:1) were added into digestion containers (glass material) for digesting the hair samples or standard materials, and the digestion containers were left to stand overnight. Thereafter, the thermal digestion was performed at 180°C by applying a DK-2 digital electric sand bath; after cooling to room temperature, 5 ml of hydrochloric acid solution (deionized water and concentrated hydrochloric acid mixed in a ratio of 1:1) was added for reduction. After reduction, 1 ml of potassium ferricyanide solution (purity ≥ 99.5%) was added into containers and adjusted to 10 ml with 5% of dilute hydrochloric acid solution. The standard curve was generated by spiking with different volumes of selenium national standard solution (1,000 μg/ml, GSB62469). The concentrations of Se standard solutions of the standard curve were 1.0, 2.0, 4.0, 6.0, 8.0, and 10.0 μg/L, respectively. The fluorescence values of the selenium national standard solution were measured using an AFS-933 atomic fluorescence spectrometer. Finally, the standard curve of selenium contents and fluorescence values was created. Under the same conditions, the fluorescence values of the blank, the national standard materials for human hair, and hair samples for humans were measured in sequence, and the selenium contents for human hair samples were calculated according to the standard curve. The recovery rate of selenium was 88.8–117.1%.

### Per capita gross domestic product

The data were obtained from the China Statistic Yearbook 2019–2020 (29, 30).

### Statistical analysis

SPSS 17.0 was used for statistical analysis. The normality was assessed using the Kolmogorov–Smirnov test. The hair selenium content was expressed as the median and interquartile range (IQR). The Mann–Whitney *U* test was conducted to compare the difference in hair selenium levels. Spearman's rank correlation analysis of the hair selenium level with per capita GDP was conducted. The *P* < 0.05 (two-sided) was considered statistically significant.

ArcGIS 9.0 was used for spatial analysis. The spatial description was performed by creating thematic maps. Moran's *I* was conducted for spatial autocorrelation analysis. Global spatial autocorrelation analysis was performed to explore spatial clustering of county-level hair selenium contents at the overall level. Local spatial autocorrelation analysis was conducted to explore the category and geography of spatial clustering of county-level hair selenium contents. The types of high-high (H-H) clusters indicated that the high values of hair selenium levels were clustered among neighboring counties (positive correlation), the types of high-low (H-L) clusters indicated that counties with high values of hair selenium levels were surrounded by those with low values (negative correlation), low-high (L-H) clusters indicated that counties with low values of hair selenium levels were surrounded by those with high values (negative correlation), and low-low (L-L) clusters indicated that the low values of hair selenium levels were clustered among neighboring counties (positive correlation).

## Results

### Demographic characteristics

**Figure 2:**
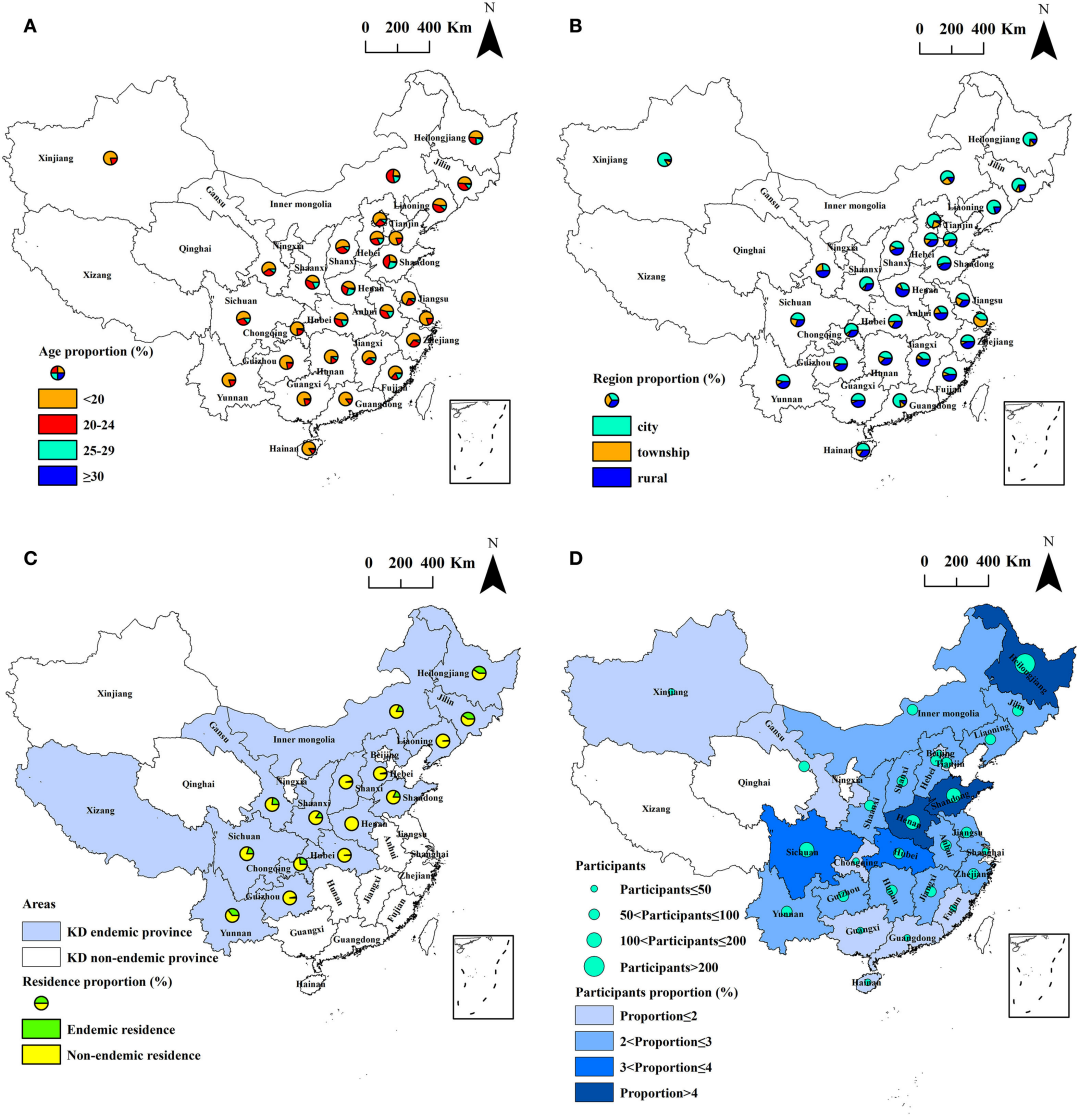
The spatial distribution of the participants by province. **(A)** Age; **(B)** region; **(C)** residence in[[Inline Image]] KD endemic province; and **(D)** participants and its proportion.

A total of 3,028 male participants aged 15–44 years participated in the study. Of the participants, 1,594 (52.7%) were aged under 20 years, 882 (29.1%) were aged 20–24 years, 479 (15.8%) were aged 25–29 years, and 73 (2.4%) were over 30 years. The number of inhabitants living in rural areas, urban centers, and cities were 843 (27.9%), 392 (12.9%), and 1,793 (59.2%), respectively. The number of inhabitants living in KD endemic and non-endemic counties were 639 (21.1%) and 2,389 (78.9%), respectively. The study regions included 1,174 counties in 28 provinces, including 15 KD endemic provinces and 13 non-endemic provinces, and covered 41.3% (1,174/2,844) of all counties in mainland China. Of the counties investigated, 170 (51.5%, 170/330) were KD endemic and 1,004 (39.9%, 1,004/2,514) were KD non-endemic. Xizang autonomous region is the only KD endemic province not participating in the study, while there is only one KD endemic county in Xizang. The spatial distribution of the participants is exhibited in Figures 2A–D.

### Hair selenium levels and KD

The hair selenium levels of the 3,028 participants from the 1,174 counties ranged between 0.04 and 22.57 mg/kg. The median of the hair selenium levels was 0.38 (IQR, 0.32–0.45) mg/kg, which was statistically significantly lower in KD endemic counties than that in KD non-endemic counties (0.34 vs. 0.39 mg/kg), *z* = −10.03, *P* < 0.0001.

**Figure 3:**
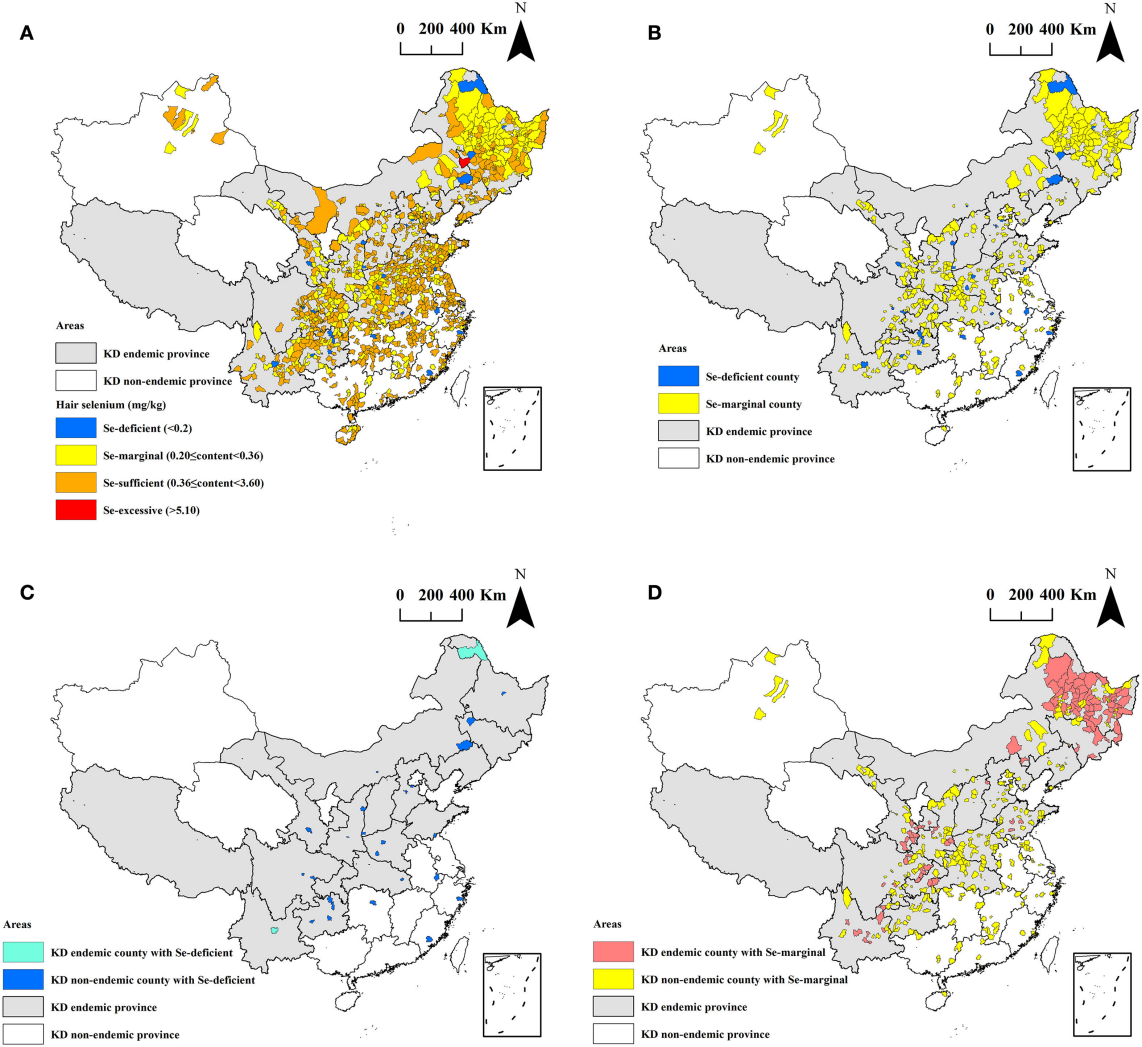
The spatial distribution of the hair selenium levels at the county level. **(A)** All 1,174 counties; **(B)** Se-deficient and Se-marginal counties; **(C)** Se-deficient counties; and **(D)** Se-marginal counties.

Of the 1,174 counties investigated, 27 (2.3%, 27/1,174) are Se-deficient, 365 (31.1%, 365/1,174) are Se-marginal, 781 (66.5%, 781/1,174) are Se-sufficient, only 1 is Se-excessive, and there is no Se-rich county. The details are presented in Figure 3A.

The spatial distribution of the Se-deficient and Se-marginal counties of the hair selenium levels is exhibited in Figure 3B. A total of 392 counties had median hair selenium levels lower than 0.36 mg/kg, accounting for 33.4% (392/1,174) of all surveyed counties in this study. Of the 392 counties, 101 are KD endemic counties, accounting for 59.4% (101/170) of all surveyed KD endemic counties, and 291 are KD non-endemic counties, accounting for 29.0% (291/1,004) of all surveyed KD non-endemic counties. The proportion of the Se-deficient and Se-marginal counties in KD endemic counties was significantly higher than that in KD non-endemic counties (*z* = −7.45, *P* < 0.0001).

The spatial distribution of the Se-deficient counties of the hair selenium levels is shown in Figure 3C. Of the 27 Se-deficient counties, 22 (81.5%, 22/27) are in KD endemic provinces, and only 5 (18.5%, 5/27) are in KD non-endemic provinces. Among the counties, 2 are KD endemic, accounting for 1.2% (2/170) of all surveyed KD endemic counties, and 25 are KD non-endemic, accounting for 2.5% (25/1,004) of all surveyed KD non-endemic counties.

The spatial distribution of the Se-marginal counties of the hair selenium levels is shown in Figure 3D. Of the 365 Se-marginal counties, 295 (80.8%, 295/365) are in KD endemic provinces, and only 70 (19.2%, 70/365) are in KD non-endemic provinces. Among the counties, 99 are KD endemic, accounting for 58.2% (99/170) of all surveyed KD endemic counties, and 266 are KD non-endemic, accounting for 26.5% (266/1,004) of all surveyed KD non-endemic counties.

### Hair selenium levels and per capita GDP

**Figure 4:**
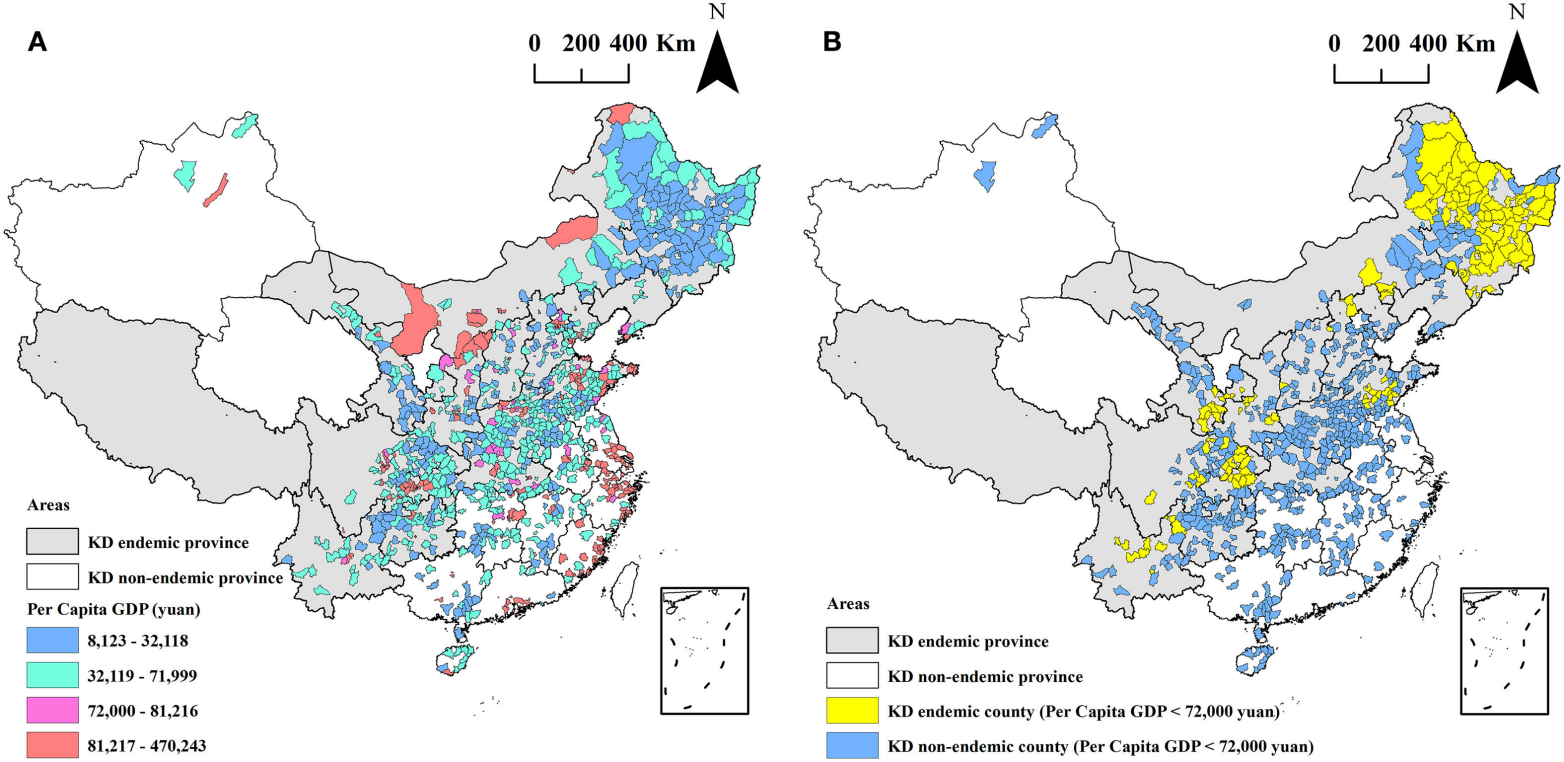
The spatial distribution of per capita GDP at the county level **(A)** and the counties with a per capita GDP lower than RMB 72,000 **(B)**.

The obtained per capita GDP of 971 out of 1,174 counties ranged between CNY ¥8,123 and CNY ¥470,243, as presented in Figure 4A. The mean of the per capita GDP was CNY ¥67,062, and it was statistically significantly lower than the mean level of the per capita GDP of China in 2020 (CNY ¥72,000). The per capita GDP of 689 counties was lower than CNY ¥72,000, accounting for 71.0% (689/971) of all collected 971 counties, as shown in Figure 4B. Among the 689 counties, 140 (94.0%, 140/149) are KD endemic, and 549 (66.8%, 549/822) are KD non-endemic. The per capita GDP of KD endemic counties was significantly lower than that of KD non-endemic counties (CNY ¥37,424 vs. CNY ¥72,434), *z* = −9.63, *P* < 0.0001. The correlation coefficient between hair selenium and per capita GDP was 0.20 (*P* < 0.0001), that is, there was a positive correlation.

### Global autocorrelation analysis of hair selenium levels

**Figure 5:**
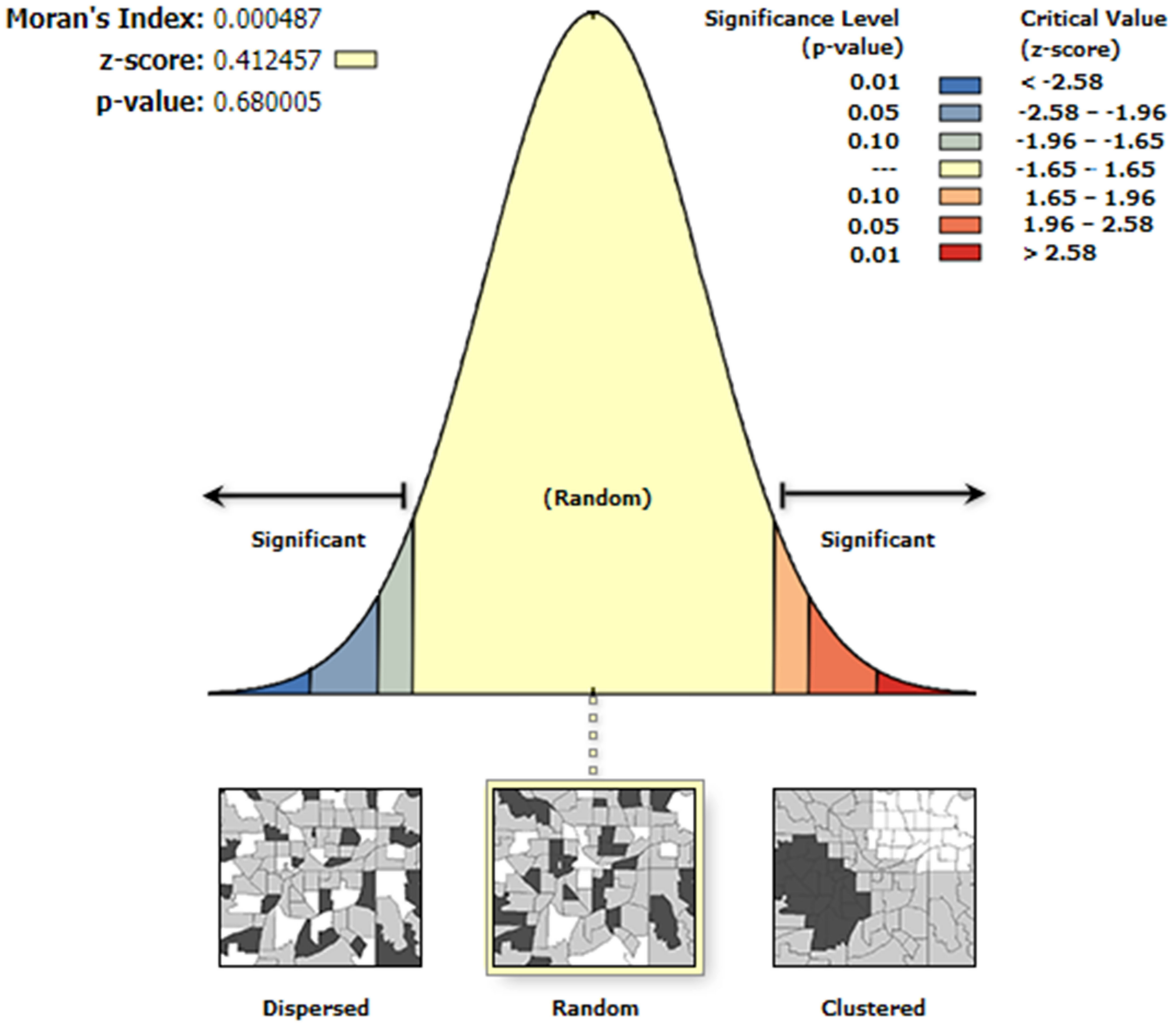
Global spatial autocorrelation analysis of the hair selenium levels at the county level.

The global autocorrelation analysis was not statistically significant (Moran's *I* = 0.0005, *P* = 0.68), indicating that there was no global clustering of hair selenium levels of the inhabitants in the 1,174 counties. The global autocorrelation analysis is shown in Figure 5.

### Local autocorrelation analysis of hair selenium level

**Figure 6:**
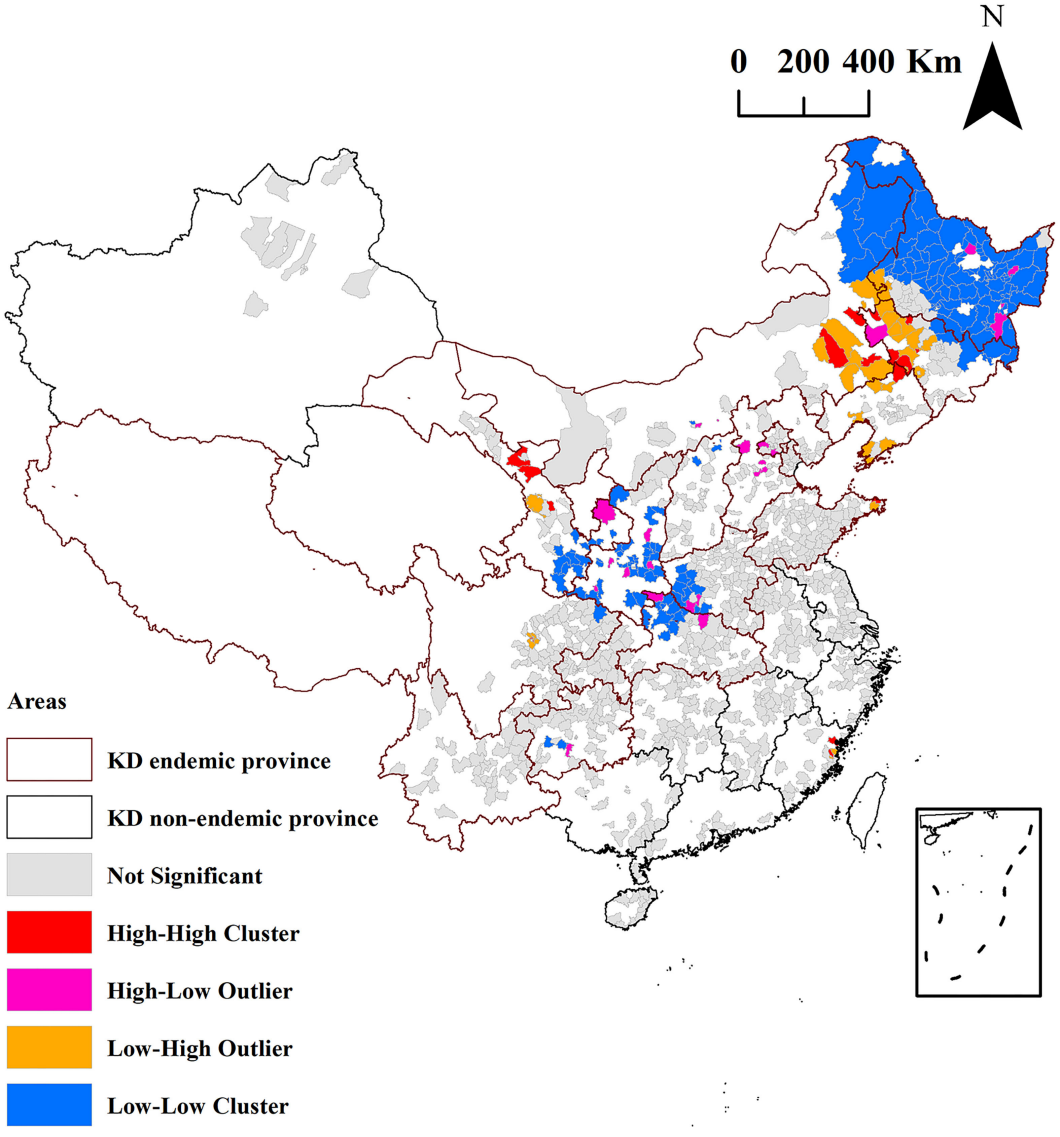
The clustered counties of hair selenium levels identified by local Moran's *I* analysis.

The local autocorrelation analysis by local Moran's *I* analysis of the hair selenium levels of the 1,174 counties is shown in Figure 6 and Table 1. The high-high (H-H) clusters of hair selenium levels were identified in 18 counties. Among the counties, 17 (94.4%, 17/18) are KD non-endemic, accounting for 1.7% (17/1,004) of all surveyed KD non-endemic counties, which are in the KD endemic provinces of Jilin, Liaoning, Inner Mongolia, Shandong, Gansu, and Sichuan and in the KD non-endemic province of Fujian; only 1 (5.6%, 1/18) is KD endemic county, accounting for 0.6% (1/170) of all surveyed KD endemic counties, which is in the KD endemic province of Jilin.

**Table 1:** The low-low clusters of the hair selenium levels of the 83 KD endemic counties identified by local Moran's *I* analysis.

**KD endemic province**	**KD endemic counties**
Heilongjiang	Yilan, Fangzheng, Bin, Bayan, Mulan, Tonghe, Acheng, Shangzhi, Wuchang, Meilisi, Yian, Gannan, Fuyu, Keshan, Kedong, Baiquan, Nehe, Didao, Jidong, Hulin, Mishan, Suibin, Lingdong, Sifangtai, Jixian, Baoqing, Raohe, Lindian, Jiayin, Tieli, Jiao, Huanan, Huachuan, Tangyuan, Fujin, Taoshan, Qiezihe, Boli, Dongning, Linkou, Suifenhe, Hailin, Ningan, Aihui, Nenjiang, Xunke, Sunwu, Beian, Wudalianchi, Lanxi, Qinggang, Qingan, Mingshui, Suiling, Hailun, Huma
Jilin	Shulan, Yanji, Dunhua, Hunchun, Wangqing
Inner Mongolia	Arunqi, Morin Dawa Daur, Oroqen, Zhalantun
Shaanxi	Yongshou, Bin, Xunyi, Baota, Shangzhou, Luonan
Gansu	Qincheng, Beidao, Zhangjiachuan, Chongxin, Huating, Jingning, Xifeng, Wudu, Xihe, Li, Hui
Henan	Luoning

The high-low (H-L) clusters of hair selenium levels were identified in 26 counties. Among the counties, 22 (84.6%, 22/26) are KD non-endemic, accounting for 2.2% (22/1,004) of all surveyed KD non-endemic counties, which are in the KD endemic provinces of Heilongjiang, Jilin, Inner Mongolia, Hebei, Henan, Hubei, Shaanxi, Gansu, and Guizhou and in the KD non-endemic provinces of Beijing and Zhejiang; only 4 (15.4%, 4/26) are KD endemic, accounting for 2.4% (4/170) of all surveyed KD endemic counties, which are in the KD endemic provinces of Heilongjiang and Shaanxi.

The low-high (L-H) clusters of hair selenium levels were identified in 37 counties. Among the counties, 33 (89.2%, 33/37) are KD non-endemic, accounting for 3.3% (33/1,004) of all surveyed KD non-endemic counties, which are in the KD endemic provinces of Heilongjiang, Jilin, Liaoning, Inner Mongolia, Shandong, Gansu, and Sichuan and in the KD non-endemic province of Fujian; only 4 (10.8%, 4/37) are KD endemic, accounting for 2.4% (4/170) of all surveyed KD endemic counties, which are in KD endemic provinces of Heilongjiang, Jilin, and Inner Mongolia.

The low-low (L-L) clusters of the hair selenium levels were identified in 174 counties. The 174 counties are all in KD endemic provinces. Among the counties, 91 (52.3%, 91/174) are KD non-endemic, accounting for 9.1% (91/1,004) of all surveyed KD non-endemic counties, which are in the KD endemic provinces of Heilongjiang, Jilin, Inner Mongolia, Shanxi, Shaanxi, Gansu, Henan, Hubei, Sichuan, and Guizhou; 83 (47.7%, 83/174) are KD endemic, accounting for 48.8% (83/170) of all surveyed KD endemic counties, which are in the KD endemic provinces of Heilongjiang, Jilin, Inner Mongolia, Shaanxi, Gansu, and Henan, as presented in Table 1.

## Discussion

This is a small area study as it was the first national spatial epidemiological study on hair selenium and KD at the county level. A small area study has the strength of geographical precision and reliable visualization because of the small size of spatial study units. The outcomes of the county-level spatial epidemiological study of hair selenium and KD were more reliable and geographically precise and visualized evidence for KD precision prevention and control at the county level from the etiological perspective, although a spatial analysis at the provincial level has been performed by our research group. Therefore, this study helps to prioritize the keys to KD precision prevention and control at the geographically smaller county level.

Selenium deficiency constitutes the most recognized evidence in terms of KD etiology (16, 19). This is well known and recognized by the World Health Organization, which classifies Keshan disease into the category of “E59, Dietary Selenium Deficiency, including Keshan Disease” in the International Statistical Classification of Diseases (ICD-10) (31). As selenium deficiency is strongly associated with KD, it does not indicate that it is the complete cause and the only cause of Keshan disease. Therefore, the indicators of selenium nutrition from the etiological perspective are more significant for primary prevention and for accessing the achievement of KD prevention and control than the indicators of the incidence or prevalence, as carried out by the public health sector. As displayed in Figures 1, 2, a total of 3,028 inhabitants from 1,174 counties in 28 provinces were included in this study. The surveyed counties covered 41.3% (1,174/2,844) of all counties in mainland China. Of the counties investigated, 170 (51.5%, 170/330) were KD endemic and 1,004 (39.9%, 1,004/2,514) were KD non-endemic. The scope of the study is wide.

The median of the hair selenium levels of the 3,028 inhabitants in the 1,174 counties was 0.38 mg/kg. It was higher than the median of the hair selenium levels of 0.33 mg/kg reported in our previous spatial study of KD and hair selenium at the provincial level (32). The median of the hair selenium contents of KD endemic counties in this study was also higher than the results reported in a previous study (0.34 vs. 0.30 mg/kg) (32). This result suggests that the selenium nutritional status of the internal environment of the inhabitants living in the KD endemic counties is improving. Globally, the median of the hair selenium levels of 0.38 mg/kg in the present study was also lower than the results of healthy Greek [0.42 mg/kg (33)], Indian [0.78 mg/kg (34)], and Hualien inhabitants in Taiwan [Han: 0.70 mg/kg, aboriginal inhabitants: 0.57 mg/kg (35)] and higher than the results of northern Polish [0.30 mg/kg (36)], Russian [0.30 mg/kg (37)], and Czech Republic [0.27 mg/kg (38)] inhabitants. Hou et al. (2) reported that the Se levels of rice and flour in KD endemic areas were higher than that of corn. In the past 40 years, inhabitants living in KD endemic areas changed their staple diet to more flour and rice instead of more corn and dried sweet potatoes; increased their intake of meat, eggs, and fish; and decreased their consumption of self-produced grains (39). Furthermore, the improving selenium nutritional status may be related to the growth of income, which greatly changed the dietary structure of residents and allowed people to consume many kinds of foods (40). Therefore, these findings suggest that an increase in the selenium nutritional status of the inhabitants living in the KD endemic counties may be dependent on the increase in the intake of a balanced diet and foods rich in selenium.

As presented in Figure 3, the hair selenium levels of 392 counties were in Se-deficient and Se-marginal status. Of the 392 counties, 101 are KD endemic counties, accounting for 59.4% (101/170) of all surveyed KD endemic counties. The proportion of the Se-deficient and Se-marginal counties in KD endemic counties was significantly higher than that in KD non-endemic counties. Furthermore, the median of the hair selenium contents of KD endemic counties was statistically significantly lower than that of KD non-endemic counties. These results suggest that the Se-deficient or Se-marginal status of the external environment of some KD endemic regions may still exist. The food chain is an important source of human intake of selenium, but the selenium contents of food are highly susceptible to the selenium levels in the external environment (3). Therefore, the Se-deficient or Se-marginal status of the external environment consequently led to insufficient selenium intake through the food chain (41, 42). Consequently, the inhabitants of the KD endemic regions may still be at risk of having KD. Thus, it is of great significance for the public health sector to monitor the selenium nutritional status of the internal and external environment in KD endemic regions. Furthermore, it would be of great importance to recommend selenium-rich diets in KD-endemic areas.

The per capita GDP of 71.0% (689/971) counties in this study was lower than the mean level of the per capita GDP of China in 2020 (CNY ¥72,000), as shown in Figure 4B. Among the 689 counties, 140 are KD endemic counties, accounting for 94.0% (140/149) of all collected KD endemic counties. The mean level of the per capita GDP in KD endemic counties was significantly lower than that in KD non-endemic counties. These results indicate that KD was closely correlated with economic factors. The results of the spatial regression showed that the hair selenium content featured a positive correlation with per capita GDP. This was consistent with the results of local autocorrelation analysis, which indicated that the low-low clusters of hair selenium levels were identified in 83 KD endemic counties with poor economic conditions. Furthermore, other studies investigating the relationship between selenium and endemic diseases related to its deficiency, such as Kashin-Beck diseases (KBD), reported that insufficient and unbalanced intakes of nutrients might also play important roles in the development of KBD (43, 44). Moreover, it is well confirmed that nutrient intake is closely associated with income levels, which tend to increase with higher income (45, 46). Therefore, it is worth thinking that the positive correlation between hair selenium and per capita GDP might involve other nutrients instead of selenium only. Further studies are needed to clarify this issue. Furthermore, the result of the prevalence of KD decreases with the increased hair selenium level found by Zhang et al. (32). These results support the etiological evidence from the etiological perspective of the remote cause of the causal chain of KD.

As shown in Figure 6, the L-L clusters of the hair selenium levels were identified in 174 counties using local Moran's *I* analysis. Among the 174 counties, 83 (47.7%, 83/174) are KD endemic, accounting for 48.8% (83/170) of all surveyed KD endemic counties. The results of the local Moran's *I* analysis at the county level were more reliable and geographically precise for KD precision prevention and control than the results of a spatial study of KD and hair selenium at the provincial level reported by our team because of its smaller size of spatial study units (32). The L-L clusters indicated that the low values of hair selenium levels were clustered among neighboring counties (positive correlation). Thus, the L-L clusters of hair selenium levels of the 83 KD endemic counties identified by local Moran's *I* analysis should be the geographical evidence for the high priority of KD precision prevention and control at the level of selenium nutrition in terms of etiology. They were of great significance in primary prevention and public health.

The major findings of the study are as follows. First, this was the first national county-level spatial epidemiological study of hair selenium and KD. Second, this study is a reliable and geographically precise small-area study. Third, the results of spatial analysis are not only the evidence for evaluating the achievement of KD prevention and control at the level of selenium nutrition in terms of etiology but also the geographically precisely visualized evidence for the high priority for KD prevention and control. Briefly, this study is a case of the public health sector translating small-area studies and selenium nutrition into the practice of KD precision prevention and control. The limitations of this study are as follows. First, this study was not probability sampling. Second, the subjects of the study were all male inhabitants. It is difficult to collect hair samples from female participants because of the impact on their appearance and hair perming or dyeing, which is very common among female participants in mainland China. Furthermore, women usually have somewhat different diet habits, i.e., less meat, etc., and therefore could be even more at risk of selenium deficiency or may show different patterns across the counties. Third, the questionnaire did not include information about regular foodstuffs.

In conclusion, the median of the hair selenium levels of inhabitants living in KD endemic counties was statistically significantly lower than that in KD non-endemic counties. The median of the hair selenium levels of inhabitants in 59.4% of KD endemic counties was in Se-deficient or Se-marginal status. The low-low clusters of hair selenium levels of the 83 KD endemic counties identified should be given high priority in KD precision prevention and control. These findings are the geographically precise and visualized evidence for the assessment of the effectiveness of KD prevention and control at the level of selenium nutrition in terms of etiology.

## Data availability statement

The original contributions presented in the study are included in the article/supplementary material, further inquiries can be directed to the corresponding authors.

## Ethics statement

The studies involving human participants were reviewed and approved by the Ethics Committee of the Harbin Medical University (HMUECDC 20180301). All participants provided written informed consent. Written informed consent to participate in this study was provided by the participants' legal guardian/next of kin.

## Author contributions

YJ, QL, and TW: formal analysis, methodology, visualization, writing—original draft, and writing—review and editing. YJ, GL, RW, CF, LQ, YuW, SS, YZo, XL, YZh, LD, HS, and SH: data curation, investigation, software, and validation. JH and HF: supervision. QL and TW: conceptualization, funding acquisition, project administration, and supervision. All authors read and approved the final manuscript.

## Funding

This study was supported by the National Natural Science Foundation of China (Grant Numbers 82073492, 81773368, 81202154, and 81172607).

## Conflict of interest

The authors declare that the research was conducted in the absence of any commercial or financial relationships that could be construed as a potential conflict of interest.

## Publisher's note

All claims expressed in this article are solely those of the authors and do not necessarily represent those of their affiliated organizations, or those of the publisher, the editors and the reviewers. Any product that may be evaluated in this article, or claim that may be made by its manufacturer, is not guaranteed or endorsed by the publisher.
